# The a-peel of storing mature seeds for wild banana conservation

**DOI:** 10.1093/conphys/coac049

**Published:** 2022-07-22

**Authors:** Emma L Dalziell

**Affiliations:** School of Biological Sciences, University of Western Australia, Perth, Western Australia, Australia and Kings Park Science, Department of Biodiversity Conservation and Attractions, Perth, Western Australia, Australia

Bananas and plantains are some of the world’s most important crops but are susceptible to a range of diseases. To improve their resistance to disease, scientists breed these crops (and others) with related species that have not been domesticated, which increases their genetic diversity and improves their disease resistance. However, many of these crop wild relatives are increasingly under threat in the wild. So, storing their seeds in seed banks is one way to ensure ongoing access to plant material.

Problem solved?

Not really.

Apparently, scientists determined that wild banana seeds tend to die quickly when stored in the bank, creating a real conservation problem. Simon Kallow and colleagues started investigating and figured out that fruit maturity and ripeness play a significant role in determining how well wild banana seeds fare in the bank. This was one big step towards safeguarding bananas and plantains for global food security and biodiversity conservation.

The lifespan (or longevity) of most seeds can be increased by carefully drying them and then storing them under cold conditions. However, being able to store seeds in this way relies on the seeds surviving both the drying and cooling processes in the first place. Not all seeds can do this. There can also be significant variation in expected lifespan between batches of the same species of seeds. This can be due to seed maturity within and between batches, genetic differences between mother plants or the environmental differences between collection locations.

Given these issues storing banana seeds, Kallow and his team set out to determine whether desiccation tolerance or maturity mattered the most for seed bank survival. They used two wild banana species (*Musa accuminata* and *Musa balbisiana*) collected from plantations or gardens across the tropics in Guadeloupe, The Philippines and Vietnam and conducted experiments on fruit collected at different levels of maturity and ripeness.

Firstly, the team assessed the ability of mature *M. accuminata* seeds to withstand drying to different levels, equivalent to what the seeds could experience when stored in a seed bank. Thankfully, the seeds survived this process and showed similar levels of germination when compared with seeds that were not dried. Given their success in the first experiment, the team then tested the ability of less mature *M. balbisiana* seeds to survive desiccation. But these immature seeds rapidly lost their germination ability when compared with fully mature seeds. Kallow’s team observed a similar response when comparing seeds from ripe and unripe fruit. But they wanted to dig deeper. So, they used x-ray computed tomography to understand what was happening *inside* the seeds that could explain these issues. Did the seed change its structure during the drying process? Indeed, the less mature seeds shrank during drying, which may contribute to their germination issues.

So, Kallow and team found that mature seeds of *M. accuminata* and *M. balbisiana* can tolerate drying, but immature seeds lose viability quickly when dried—possibly because the structure of the embryo is incomplete at this stage.

This discovery is truly a-peeling for banana seed collectors. Paying close attention to fruit ripeness and maturity when selecting bunches of bananas to use for conservation seed banking will result in much higher success rates. Further work is still required to determine the impact of storage temperature and duration on banana seed viability. But, for now, we know that mature seeds will have the best chance of surviving the rigours of the bank.

**Illustration:** Erin Walsh, ewalsh.sci@gmail.com


